# Home numeracy experiences are associated with number-related brain activity and connectivity in early childhood

**DOI:** 10.1038/s41539-026-00419-5

**Published:** 2026-04-03

**Authors:** Cléa Girard, Léa Longo, Hannah Chesnokova, Justine Epinat-Duclos, Jérôme Prado

**Affiliations:** 1 Laboratoire de Psychologie et Neurocognition (LPNC), CNRS UMR 5105, Université Grenoble Alpes, Grenoble, France; 2 Centre de Recherche en Neurosciences de Lyon (CRNL), INSERM U1028—CNRS UMR5292, Université de Lyon, Bron, France

**Keywords:** Neuroscience, Psychology, Psychology

## Abstract

Children vary widely in numerical knowledge before school entry, and these early differences predict later achievement. Although home numeracy experiences relate to young children’s skills, it remains unclear how early experiences shape neural systems supporting number processing at the start of formal schooling. Using fMRI, we measured brain activity during passive perception of digits (vs. letters) in 37 five-year-olds. Parents reported the frequency of home numeracy practices and engaged in a free play session allowing us to quantify number talk. Children showed digit-specific activity in the left intraparietal sulcus (IPS). Across families, higher home numeracy experiences were associated with lower digit-related activity in several regions, including the IPS, but with stronger functional connectivity between the left IPS and other regions. Our results suggest that home numeracy experiences may support early number-related brain networks by enhancing connectivity and reducing local processing demands, illustrating how home experiences influence the developing learning brain.

## Introduction

Early childhood is an important period for cognitive development, with long-lasting effects on academic learning. Even before first grade, there is significant variability in the quality and quantity of literacy and numeracy experiences children are exposed to, particularly within their home environment^1–5^. These experiences not only contribute to disparities in early literacy and numeracy skills at school entry^1,6^, but they also have long-term implications. Indeed, numerous studies have found that early academic skills are strongly related to later academic success^7^.

How do early experiences affect children’s cognitive development? Although the mechanisms underlying the relation between early experiences and cognitive development may include a domain-general component (e.g., executive functions^8^), recent neuroimaging studies predominantly suggest domain-specific pathways. For example, variability in early language experiences at home has been linked to the development of language-related brain regions^9^ through variations in white matter organization^10–12^, functional connectivity^13,14^, and language-related brain activity^15,16^.

However, the neurocognitive mechanisms underlying the relation between early numeracy experiences and children’s numerical skills are much less understood than those related to literacy experiences. This is despite the fact that several previous studies have found that the quality of the home numeracy environment, often assessed through questionnaires and linguistic analysis of caregiver-child verbal interactions, relates to numerical skills in young children^1^. For example, in a recent study^17^, we measured a wide range of mathematical skills in 128 5-year-old children, while one of their parents completed an extensive questionnaire evaluating the frequency of home numeracy practices. Parents were also asked to engage in a free play session with their child in the laboratory, allowing us to measure the type and quantity of number-related verbal input that children receive (i.e., the so-called “number talk”^18^). Although we did not replicate the previously reported association between children’s skills and parental number talk^19^, we found a relation between symbolic mathematical skills and numeracy practices that were the most formal (i.e., when parents explicitly intend to teach math concepts) and relatively advanced (i.e., above expectations for a kindergartner), in keeping with several other studies^20–23^.

To our knowledge, no study has specifically investigated the impact of early numeracy experiences on brain function. In a previous study conducted on older children (i.e., 8-year-olds)^24^, we found a relation between the frequency of numeracy activities at home and brain responses to digits in a region that is central to the representation of numerical magnitudes, i.e., the intraparietal sulcus (IPS)^25^. Yet, these results may not fully inform our understanding of the relation between numeracy experiences and the development of the numerical brain in young children for at least three reasons. First, the environment of an 8-year-old is heavily influenced by several years of formal schooling, which makes it difficult to disentangle the effects of the home environment from the co-occurring influence of formal math instruction. Second, the nature of math activities that families typically engage in with elementary-aged children differs significantly from those conducted during preschool or kindergarten. For example, a focus at these ages may be promoting the acquisition and understanding of numerical symbols. This, in turn, may support school readiness^26^. Finally, parents might play a lesser role in their children’s mathematical development during elementary school than during preschool or kindergarten, where learning is less focused on formal academics. This is supported by meta-analyses showing that the association between the home numeracy environment and children’s numerical skills is stronger in preschool than in elementary school^1,27^.

To shed light on how early numeracy experiences may impact young children’s numerical development, the objective of the present study was to examine the relation between these early experiences and the development of the brain systems supporting number processing at the very beginning of formal schooling (i.e., around 5 years of age). Our sample consisted predominantly of French children in kindergarten (though some were in preschool and at the very beginning of 1st grade). In France, compulsory education starts at around 3 years of age, with children attending two years of preschool before kindergarten in nursery schools that are called “école maternelle”. While these nursery schools follow a national curriculum (ensuring homogeneity in instruction), it primarily emphasizes play-based activities focused on socialization, language development, and motor skills. As children progress to kindergarten, there is a gradual introduction of more structured learning activities, including basic numeracy. However, formal math instruction typically starts in first grade, which provides a unique opportunity to examine the role of home numeracy experiences while minimizing the influence of formal math instruction. In the present study, we invited preschool children who participated in a prior study about the early home numeracy environment^17^ to attend an additional fMRI scanning session. Parents completed an extensive questionnaire evaluating the frequency of numeracy practices shared with children at home. Parents also engaged in a free play session with their child in the laboratory, allowing us to measure number-related verbal input that children may receive (i.e., number talk). Children’s numeracy skills were also assessed using a comprehensive battery of standardized tests. Finally, fMRI activity associated with number processing was measured using a passive viewing task (adapted from a previous study in 8-year-olds^24^ in which children completed separate runs of digit and letter blocks interleaved with fixation).

We made the following series of pre-registered hypotheses. Our first and main hypothesis was that the quality of the early home numeracy environment (measured by the frequency of home numeracy practices and instances of number talk) would be positively associated with digit-related brain activity in the IPS (hypothesis #1). Indeed, while digit processing engages brain circuits distributed across various cortical regions^28,29^, digit-related activity in the IPS may serve as a key marker of children’s numerical development. For example, IPS activity during numerical processing has been shown to increase with children’s age^30,31^ and skill level^24,32^. Connectivity between the IPS and other brain regions has also been observed during digit processing^33^. Furthermore, it has been suggested that numerical development in children is characterized by a functional shift from the right IPS—primarily involved in processing non-symbolic quantities^34–36^—towards the left IPS, as children gain experience with symbolic numerical information^37–39^. Therefore, digit processing may be particularly associated with the left IPS, even in relatively young children^28^.

Our second hypothesis was that number-specific activity in the IPS would be positively related to children’s numerical skills (hypothesis #2). Our third hypothesis was that number-specific activity in the IPS would explain the relation between the home numeracy environment and children’s numeracy skills, as observed in our previous study (hypothesis #3). Our fourth expectation, based on our previous findings^24^, was that the relation between the home numeracy environment and number-specific activity would be strongest for numeracy practices that were both the most formal and the most advanced, as well as for the type of number talk involving more frequent use of large number words^18,40^ (hypothesis #4). Our fifth hypothesis was that any relation between the home numeracy environment and IPS activity would be specific to digit processing as opposed to letter processing (hypothesis #5). Finally, our prediction was that the above relations would hold even when controlling for variables that may be potential confounding factors for the observed associations^41^ (hypothesis #6). Specifically, family SES and children’s IQ were included as general indicators of environmental and cognitive resources^42,43^. Parental numeracy skills were also controlled for because prior studies have shown that relations between home learning environments and children’s academic skills may partly reflect genetic or familial influences rather than purely environmental effects^44^. Finally, following our previous work in older children^24,41^, we controlled for parents’ subjective estimates of their child’s numeracy skills. Indeed, parents who perceive their child as more skilled might engage in more numeracy activities with them^45,46^. Controlling for perceived child ability may therefore ensure that the observed relations between home numeracy experiences and brain measures are not only explained by parents adapting their practices to their child’s perceived skills.

## Results

### Behavioral associations between children’s numeracy skills and home numeracy practices

A comprehensive report of associations between children’s numeracy skills and home numeracy practices in the entire sample of children who participated in the first behavioral session can be found in our previous report^17^. Briefly, we found in the original sample (*n* = 128) a positive association between advanced formal home numeracy practices and children’s transcoding and calculation skills (controlled for basic formal practices). However, as also reported in Girard et al.^17^, we did not observe any significant association between children’s numeracy skills and either basic formal practices or informal home numeracy practices.

**Fig. 1:**
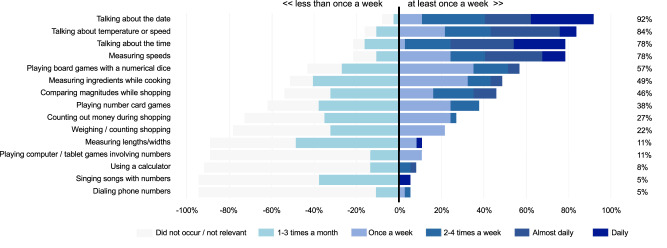
Divergent stacked bar chart showing the frequencies of informal practices among parents. Percentages on the right indicate the share of parents who engaged in each practice at least once a week. Practices are ordered from the most frequent (top) to the least frequent (bottom).

**Fig. 2:**
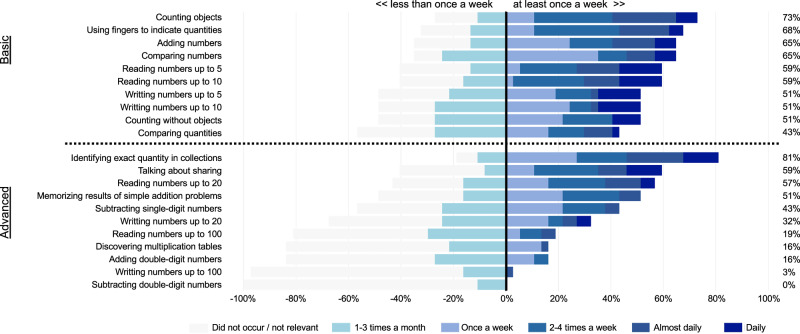
Divergent stacked bar chart showing the frequencies of basic and advanced formal practices among parents. Percentages on the right indicate the share of parents who engaged in each practice at least once a week. Practices are ordered from the most frequent (top) to the least frequent (bottom), separately for each level of complexity.

To evaluate whether these findings held in the subsample of children who were included in the fMRI analyses (*n* = 37), we performed the same analyses in this subsample. First, similar to the full sample, a qualitative examination of reported home numeracy practices indicated wide variability in this subsample (see Figs. 1 and 2). Among parents, mean responses on the rating scale ranged from 0.20 to 2.80 for informal practices (*M* = 1.51, *SD* = 0.49), from 0.10 to 4.40 for basic formal practices (*M* = 2.12, *SD* = 1.17), and from 0 to 2.73 for advanced formal practices (*M* = 1.23, *SD* = 0.69).

Correlational analyses in the fMRI subsample replicated key findings from the original sample (see Table S1 for the full correlation matrix, including significance levels and Bonferroni correction). Controlling for basic formal practices, we found a positive relation between advanced formal practices and children’s transcoding and calculation skills (transcoding: *r*(35) = 0.63, *p* < 0.001; calculation: *r*(35) = 0.40, *p* = 0.015). As in the full sample, we did not find any positive relation between advanced formal practices and most other numeracy skills (all *r*s < 0.26). One notable difference between the full sample and the fMRI subsample was observed with non-symbolic skills, which was found to be positively correlated with advanced formal numeracy practices in the fMRI subsample (*r*(35) = 0.38, *p* = 0.024). However, because this relation was not observed in the full sample^17^, it should be considered with caution. Consistent with the full sample, no significant associations were observed between children’s numeracy skills and either basic formal or informal practices in the fMRI subsample (all *r*s < 0.28). These findings are summarized in Table S2. To assess the robustness of these associations, we conducted sensitivity analyses controlling for potential confounding variables, including child sex, age, and head motion during fMRI scanning. All key findings remained significant when these covariates were taken into account (see Table S3 in the Supplementary Materials).

We then performed two exploratory (i.e., not pre-registered) analyses in the fMRI subsample. First, previous studies have found a relation between children’s numeracy skills and the number of games at home reported by parents, suggesting that this measure may act as a proxy for informal math exposure^21,22^. Therefore, we assessed the relation between the number of children’s games that parents reported having at home and children’s numeracy skills. There was a positive relation between the number of children’s games at home and both counting and non-symbolic skills (counting: *r*(35) = 0.36, *p* = 0.030; non-symbolic skills: *r*(35) = 0.50, *p* = 0.002). This pattern was relatively specific to these skills, as there was no positive relation between the number of children's games and any other skills (all *r*s < 0.27). These associations remained significant after controlling for socioeconomic status (parental education and income). Specifically, the number of children’s games at home was still positively associated with counting skills (partial *r*(35) = 0.35, *p* = 0.04) and non-symbolic skills (partial *r*(35) = 0.51, *p* = 0.002). Second, it has been argued that the relation between parental numeracy practices and children’s numeracy skills may not necessarily be unidirectional. For example, parents may also engage in numeracy practices because they respond to their child’s skills and interests. To evaluate whether this was the case, we tested whether there was a relation between the parental assessment of children’s numeracy skills and home numeracy practices. We did not find any significant relation between this subjective assessment and any subtype of home numeracy practices (all *r*s < 0.29, all *p*s > 0.09). However, there was a positive relation between the parental assessment of children’s numeracy skills and the number of children’s games at home (*r*(35) = 0.38, *p* = 0.019).

### Behavioral associations between children’s numeracy skills and parental number talk

Our previous investigation of parental number talk in the full behavioral sample did not reveal any relation between children’s numeracy skills and either total number words or subcategories of number words^17^. In the fMRI subsample (as in the full behavioral sample), there was a large variation in the total number of words spoken by parents, from 132 to 1332 words (*M* = 790, *SD* = 289). On average, there were 34 instances of number words across families, also with a large variability (*SD* = 28, range = 0–121). However, the proportion of total number words was not positively related to any numeracy skills (all *r*s < 0.07). This was also the case when only the largest number words ( > 10) were considered (all *r*s < −0.01). Thus, as in the full behavioral sample, we did not find any relation between parental number talk and our measures of numeracy skills in the fMRI subsample.

Finally, we also tested whether there was a relation between the parental assessment of children’s numeracy skills and the proportion of total number words. We did not find any significant relation (*r*(35) = −0.04, *p* = 0.795).

### Average number-related activity across all children

**Fig. 3:**
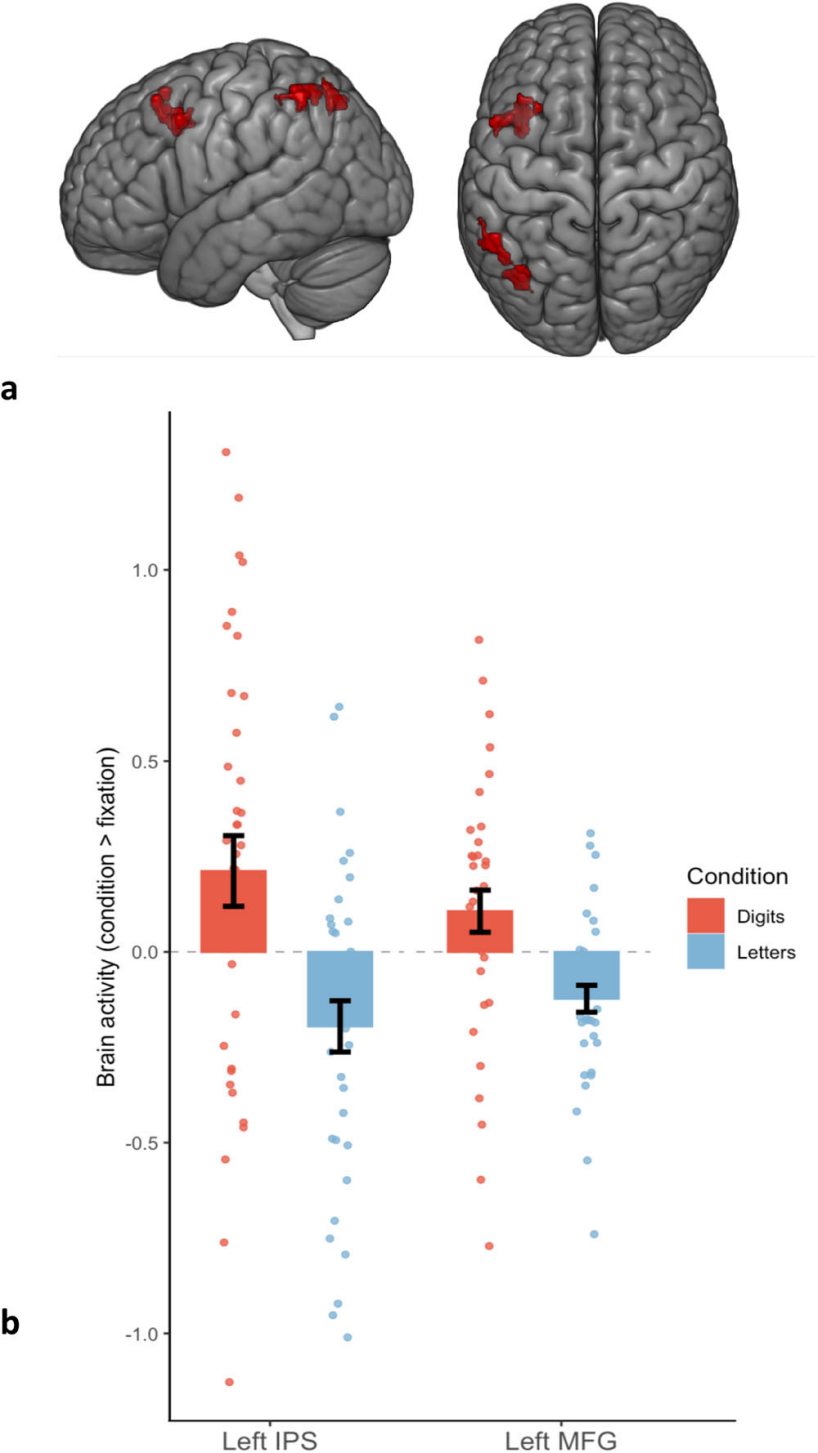
Digit-specific brain activity in the left IPS and left MFG in 5-year-old children. **a** Standard brain surface renderings showing regions with significantly greater activity for digits than for letters (Digits > Letters contrast) across all participants. The highlighted clusters are located in the left Intraparietal Sulcus (posterior) and the left Middle Frontal Gyrus (anterior), and reflect number-specific brain activity at the group level (see Results Section “Average number-related activity across all children”). These clusters survived whole-brain correction for multiple comparisons using a cluster-forming threshold of *p* < 0.0025 (uncorrected) and a cluster-level FWE correction at *p* < 0.05. **b** Mean beta values extracted from each cluster for the contrasts Digits > Rest (Red) and Letters > Rest (Blue) in the Left IPS and Left MFG across participants. Each dot represents one child; Error bars indicate the standard error of the mean.

Before turning to our pre-registered hypotheses, we first evaluated whether any brain region showed number-specific activity across all children. This was done by contrasting activity associated with blocks of digits to activity associated with blocks of letters. We found number-specific activity in two left-lateralized clusters located in the IPS (*x* = −54, *y* = -52, *z* = 52; *Z* = 4.24; volume = 1750 mm^3^) and in the Middle Frontal Gyrus (*x* = −34, *y* = 14, *z* = 48; *Z* = 3.94; volume = 1918 mm^3^) (see Fig. 3).

### Relations between the home numeracy environment and number-specific activity

As pre-registered, we hypothesized that the quality of the home numeracy environment (measured by the frequency of home numeracy practices and the instances of number talk) would be associated with brain activity, particularly in the IPS (hypothesis #1). Nonetheless, all analyses reported here were conducted using whole-brain voxelwise regressions to allow for identification of effects in the IPS but also beyond that brain region. We further anticipated that this relation would be specific to the processing of digits compared to letters (hypothesis #5).

**Fig. 4:**
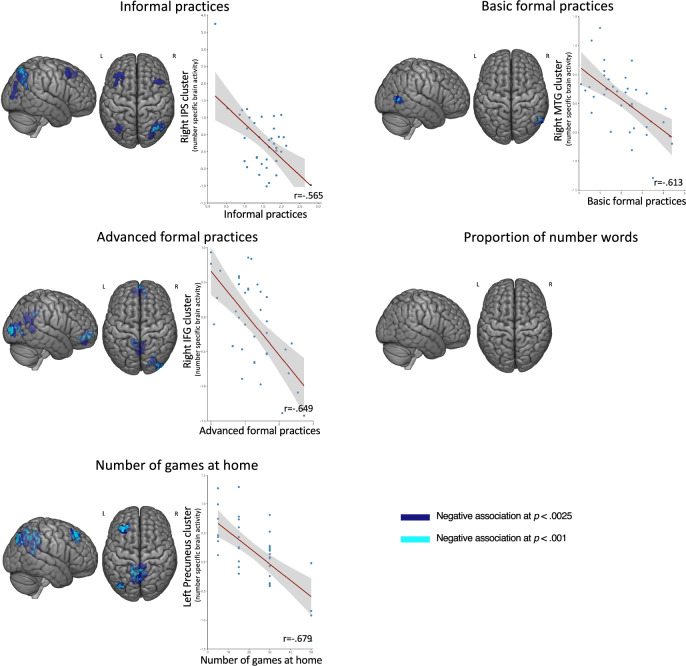
Negative associations between home numeracy experiences and number-specific brain activity. Surface renderings and scatterplots showing brain regions where number-specific activity (Digits > Letters) was negatively associated with higher levels of different aspects of the home numeracy environment. Clusters are shown in dark blue for *p* *<* 0.0025 and in light blue for *p* *<* 0.001 (both FWE-corrected at the cluster level). Scatterplots illustrate individual data points and regression lines.

In line with these hypotheses, whole-brain regression analyses confirmed that brain activity associated with perceiving digits (compared to perceiving letters) was related to both the frequency of home numeracy practices. However, contrary to our hypothesis, it was not related to the proportion of total number words after removing one outlier (Cook’s distance > 1) that was driving spurious correlations. As can be seen in Table 1 and Fig. 4, all types of home numeracy practices (informal, basic formal and advanced formal) were related to number-specific activity in several regions (see also Fig.4 for scatter plots of brain-behavior correlations in most active clusters for all relevant measures). This was also the case of the number of games parents reported having at home, which was an exploratory variable. Critically, number-specific activity in the IPS was related to the frequency of informal numeracy practices and the number of children’s games at home. However, associations with these variables clearly extended beyond the IPS and were also found in regions of the frontal as well as temporal cortices. This was notably the case for formal numeracy practices.

**Table 1:** Brain regions in which number-specific activity was negatively associated with quality of the home numeracy environment

Anatomical location	MNI coordinates			*Z*-score	*p*FWE corrected	Cluster sizeat *p* < 0.0025 (voxels)	Cluster sizeat *p* < 0.001(voxels)
	*X*	*Y*	*Z*				
Informal practices							
Left IPS	−32	−74	48	4.23	0.030	111	-
Left Superior Frontal Gyrus	−26	30	48	4.22	0.003	169	-
Right IPS*	38	−64	52	4.01	<0.001	410	217
Left Inferior Frontal Gyrus	24	−84	6	3.75	0.026	114	-
Right Middle Frontal Gyrus	40	16	22	3.63	0.039	105	-
Basic formal practices							
Right Middle Temporal Gyrus*	54	−58	2	4.70	0.033	110	65
Advanced formal practices							
Right Inferior Frontal Gyrus*	36	−90	2	4.52	<0.001	344	213
Right Precuneus*	6	−48	6	4.41	<0.001	396	94
Left Medial Frontal Cortex*	0	60	−4	3.94	<0.001	262	78
Proportion of number words							
* No significant cluster*							
Number of children’s games at home							
Left Precuneus*	−8	−56	44	5.17	<0.001	1275	775
Left Superior Frontal Gyrus*	−20	36	41	4.36	<0.001	234	171
Left IPS*	−40	−78	38	4.27	0.012	133	90

All clusters indicated in the table are significant at *p* < 0.0025 (voxelwise) and p < 0.05 FWER corrected (clusterwise).

* indicates clusters that are also significant at *p* < 0.001 (voxelwise) and *p* < 0.05 FWER corrected (clusterwise). All Z-values reflect one-sided statistical maps derived from whole-brain analyses, and are therefore presented as positive values. The direction of the associations (positive or negative) is described in the main text.

*MNI* Montreal Neurological Institute.

Another pre-registered hypothesis was that the relations above would hold even when controlling for family SES (i.e., parental education and income), children’s IQ, parental numeracy skills, and parental subjective estimate of their child’s numeracy skills (hypothesis #6). To test this hypothesis, in line with our pre-registered plan, we included all of these covariates in additional whole-brain regression models. As shown in Supplementary Fig. 1, the relations between most dimensions of home numeracy experiences and number-specific activity remained significant in the same regions, even after accounting for these variables.

Interestingly, exploratory analyses revealed that the relations above between the home environment and number-specific activity in children were relatively specific to numeracy. Indeed, as can be seen in Supplementary Fig. 2, number-specific activity was not significantly related to informal and basic formal literacy practices, overall number of words spoken by parents during the free play session, or number of books at home. The only relation found was between advanced formal literacy practices and number-specific activity in an occipital cluster.

Critically, however, several aspects of our pre-registered hypotheses were not confirmed. First, we anticipated that number-specific activity in the IPS would be more strongly linked to formal than informal dimensions of the home numeracy environment (hypothesis #4). This did not appear to be the case, as informal but not formal practices were associated with number-specific activity in the IPS (see Fig. 4). Second, we anticipated that generally the relation between the home numeracy environment and number-specific activity would be the strongest for advanced formal practices and more frequent use of large number words (hypothesis #1). As shown on Fig. 4, the relation between the home numeracy environment and number-specific activity was observed across all subtypes of numeracy practices, including informal and basic formal. Separate analyses of the relation between the proportion of parental number words and number-specific activity as a function of the magnitude of the words (i.e., small, medium, and large) did not reveal any significant clusters after removing the outlier (see above). Third, in all hypotheses we expected all relations between quality of the home numeracy environment and number-specific activity to be positive, as in our previous study^24^. In contrast to this prediction, however, higher-quality of the home numeracy environment (indexed by more frequent numeracy practices and greater number of games at home) were systematically associated with lower rather than higher number-specific brain activity.

Overall, then, we were able to confirm some but not all aspects of our pre-registered hypotheses regarding the relations between the home numeracy environment and brain activity. On the one hand, we found a relation between quality of the home numeracy environment and number-specific activity in several regions (including the IPS), controlling for a number of factors. On the other hand, that relation was found across most categories of practices, was not found for number talk, and was systematically negative rather than positive when it was found. Exploratory connectivity analyses below explore a potential explanation for why these relations might be negative.

To further explore whether the observed associations were specifically driven by digit-related activity rather than reduced letters-related activity, we conducted additional exploratory analyses using the Digits > Rest contrast extracted from the same functional clusters. These analyses revealed a qualitatively similar negative pattern of associations (Supplementary Fig. 3).

### Relations between the home numeracy environment and number-specific connectivity

A surprising and unexpected aspect of our results is the fact that the relations between the home numeracy environment and number-specific activity are negative rather than positive. In other words, a higher quality home numeracy environment (i.e., more frequent practices) is associated with lower rather than higher brain activity associated with number processing (compared to letter processing). One possibility to explain this result is that children who have the highest quality home numeracy environment might be those who have the greatest weakness in numerical processing, which would be reflected by less number-specific activity. However, this is inconsistent with our behavioral results, which showed that home numeracy practices (and number of children’s games) are associated with better numeracy skills (i.e., transcoding and calculation). This is also inconsistent with the fact that there was no negative relation between the parental assessment of children’s numeracy skills and the quality of the home numeracy environment (see above). Therefore, it is unlikely that the quality of that environment increases in response to a perceived weakness in children.

**Fig. 5:**
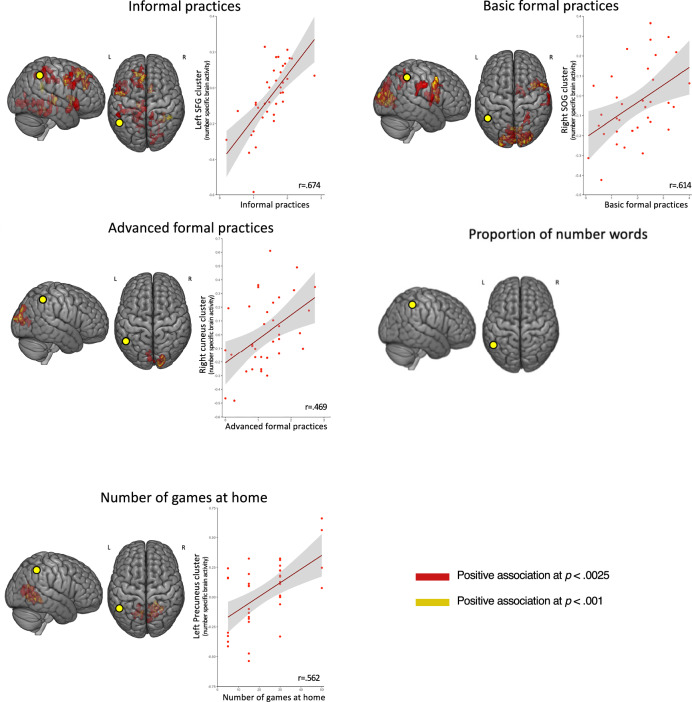
Positive associations between home numeracy experiences and number-specific functional connectivity with the left IPS. Surface renderings of brain regions showing positive associations between number-specific functional connectivity with the left Intraparietal Sulcus (shown as a yellow circle) and higher levels of different aspects of the home numeracy environment. Scatterplots display individual data points and fitted regression lines for each significant brain-behavior association. Clusters are color-coded according to the statistical threshold (red for *p* < 0.0025 and yellow for *p* < 0.001).

Another possibility is that lower number-specific activity reflects greater efficiency in the cognitive system, perhaps because regions become more connected with higher quality home numeracy environment. Indeed, greater communication between regions may decrease local processing demands^47,48^ and changes in functional connectivity may be more prominent than changes in functional activity in young children^49^. To test this hypothesis, we used PPI to measure whether communication between the IPS and other brain regions during number processing (compared to letter processing) was stronger in children exposed to higher quality of the home numeracy environment. The IPS seed region was defined based on the contrast of numbers versus letters across all children, which showed activation in a left-lateralized cluster (see Fig. 3). We found that number-specific connectivity between that left IPS cluster and several prefrontal, parietal, and occipital regions was positively associated with the frequency of all subcategories of home numeracy practices, as well as for the number of children’s games at home (see Table 2, see also Fig. 5 for scatter plots of brain-behavior correlations in most active clusters for all relevant measures). Therefore, with the exception of the proportion of total number words that were not found related to number-specific connectivity, all aspects of the home numeracy environment were positively related to number-specific connectivity with the left IPS.

**Table 2:** Brain regions in which number-specific functional connectivity with the left IPS was positively associated with quality of the home numeracy environment

Anatomical location	MNI coordinates			*Z*-score	*p*FWE corrected	Cluster size at *p* < 0.0025 (voxels)	Cluster siz at *p* < 0.001(voxels)
	*X*	*Y*	*Z*				
Informal practices							
Left Superior Frontal Gyrus*	−10	24	52	5.28	<0.001	474	209
Left Precentral Gyrus*	−46	0	38	5.07	<0.001	231	100
Left Thalamus*	−8	−26	−4	4.71	0.009	138	80
Left Lingual Gyrus	-12	-42	-4	4.60	0.009	139	-
Right Basal Ganglia*	6	8	−12	4.43	0.037	104	61
Right Precuneus	10	−40	58	4.14	0.039	103	-
Left Precuneus	−14	−40	58	4.11	0.001	186	-
Left Fusiform Gyrus	−32	−30	−18	4.10	0.011	133	-
Left Anterior Insula	-38	22	−12	4.08	0.009	137	-
Left IPS	−40	−32	38	4.04	0.021	117	-
Right Fusiform Gyrus*	34	−42	−12	3.89	0.004	160	65
Right Lingual Gyrus	12	−86	−1	3.86	0.003	163	-
Basic formal practices							
Right Superior Occipital Gyrus*	18	−86	16	4.52	<0.001	1483	734
Right Inferior Frontal Gyrus*	48	8	10	4.33	<0.001	364	168
Right IPS	24	−48	44	4.00	0.010	139	-
Right Supramarginal Gyrus	64	−22	30	3.98	0.035	108	-
Right Thalamus	8	−6	10	3.79	0.021	120	-
Advanced formal practices							
Right Cuneus*	10	−90	20	3.93	.<0.001	233	125
Left Cuneus	−6	−86	13	3.71	0.034	111	-
Proportion of number words							
*No significant cluster*							
Number of children’s games at home							
Left Precuneus*	−6	−58	6	4.21	0.001	215	80
Right Lingual Gyrus*	16	−52	−4	4.17	<0.001	484	202

All clusters indicated in the table are significant at *p* < 0.0025 (voxelwise) and *p* < 0.05 FWER corrected (clusterwise). * indicates clusters that are also significant at *p* < 0.001 (voxelwise) and *p* < 0.05 FWER corrected (clusterwise). All Z-values reflect one-sided statistical maps derived from whole-brain analyses, and are therefore presented as positive values. The direction of the associations (positive or negative) is described in the main text.

*MNI* Montreal Neurological Institute.

### Relations between number-specific activity and children’s numeracy skills

Another pre-registered hypothesis was that number-specific activity in the IPS would be related to children’s numeracy skills (hypothesis #2). Contrary to this hypothesis, our whole-brain analyses failed to reveal any significant relation between number-specific activity and any of the children’s numeracy skills anywhere in the brain (at least at the statistical threshold chosen). Therefore, we were also unable to formally test our subsequent hypothesis that number-specific activity in the IPS would account for the relation between the home numeracy environment and children’s numeracy skills (hypothesis #3). We speculate on the possible reasons for the lack of significant relation between number-specific activity and children’s numeracy skills in the discussion.

## Discussion

The present study aimed to explore the relation between early home numeracy experiences and number-related brain function in young children. Our main findings are twofold. First, they reveal that the passive perception of digits (compared to letters) is associated with enhanced activity in a left-lateralized fronto-parietal system in 5-year-old children, including the IPS. Second, they show that differences in home numeracy experiences at that age (measured through parental reports of home numeracy practices and measures of number talk) are associated with differences in levels of activity and functional connectivity with the IPS in several prefrontal, parietal, and occipital regions.

As described in “Material and Methods Section” “Pre-registration and justification of sample size”, we initially planned to assess number-specific brain activity by examining neural adaptation to repeated symbolic stimuli, based on our previous study in older children^24^. However, because no robust adaptation effects were observed in the present younger sample of 5-year-olds, we instead focused on a between-category contrast (digits vs letters). Similar contrasts have been previously used to study emerging symbolic number processing in preschool-aged children^50^. Indeed, such contrasts may not require the level of automaticity needed to elicit robust neural adaptation effects.

As shown in Section “Average number-related activity across all children” and Fig. 3, the passive perception of digits (compared to letters) was associated with enhanced activity in both the left IPS and the left Middle Frontal Gyrus across all children. These findings indicate a selective recruitment of parietal and frontal regions during symbolic number perception at this relatively young age. Activity in the frontal cortex is notably consistent with previous studies that have observed greater involvement of frontal regions in children than adults^51,52^. This may indicate a greater reliance on working memory and executive functions when these newly learned symbols are not yet automatized^53^. It may also indicate that frontal mechanisms might be involved in the association between symbols and their semantic meanings^54–56^. To some extent, the involvement of the left IPS in our sample of 5-year-old children may be more notable because the recruitment of left IPS mechanisms during numerical processing is often thought to be contingent upon formal learning and to develop gradually. This is suggested by studies demonstrating a correlation between left IPS activity during symbolic processing tasks and either age or participant performance^32,57,58^. Though some studies have failed to observe left-lateralized IPS activity in young children in symbolic comparison tasks^59^, our results are consistent with other studies that did show early engagement of the left IPS in matching or symbolic comparison tasks^37,60^. Therefore, the present study suggests that left IPS mechanisms are sensitive to symbolic numerical information relatively early in development. This sensitivity may be further enhanced as children become proficient in using these symbols. This is supported by the fact that sequentially repeating the same number (compared to repeating different numbers) was not associated with decreased activity in the left IPS, whereas that effect was present in older children in a previous study^24^. Therefore, although 5-year-old children may recognize that digits represent quantities, accessing quantitative information from digits may not be fully automatized at that age^61^. Importantly, however, behavioral results confirmed that children in the fMRI sample accurately understood the quantitative meaning of the digits, as indicated by their near-ceiling performance on the symbolic number comparison and written numerical decision tasks. This supports the interpretation that left IPS activity in the present study may result (at least in part) from genuine symbolic number processing and may not simply reflect mere visual familiarity with number symbols.

Our main pre-registered hypothesis was that home numeracy experiences would also be positively associated with number-related brain activity in young children. We did find that activity of several brain regions was related to differences in (i) the frequency of home numeracy practices, (ii) the quantity of parental number talk measured in the free play session, and (iii) the number of children’s games at home. Unexpectedly, however, the relations were systematically negative, in contrast to the pre-registered direction of that relation. Indeed, enhanced home numeracy experiences were systematically associated with decreased levels of brain activity, contrary to our previous study in older children, in which the relation was positive^24^.

Several explanations may account for this finding. One is that home numeracy experiences might constitute a parental response to perceived struggles in children, as indexed by lower levels of activity associated with the perception of digits (compared to letters). In other words, home numeracy experiences might not cause changes in brain activity rather than the reverse. However, this explanation is inconsistent with the finding that there was a positive relation between the symbolic numeracy skills of children who participated in the present fMRI study (i.e., transcoding and arithmetic) and the frequency of home numeracy practices reported by their parents (notably practices that were formal and relatively challenging) (for consistent results with a larger sample, see Girard et al.^17^). Therefore, children who benefited from improved numeracy experiences were those with the most advanced numerical skills. This suggests another possibility to explain the negative relation between home numeracy experiences and brain activity: Decreased brain activity associated with more frequent home numeracy experiences may reflect experience-dependent tuning and increased local processing efficiency. For example, this could be because early development is often associated with an increase in communication between brain regions^49,62^, which may reduce processing demands on each individual region^48^. Such a pattern of reduced local activity coupled with increased inter-regional connectivity has been described as a hallmark of experience-induced optimization in cortical networks^47^ and would be consistent with our exploratory results on brain connectivity (see below). The idea that reduced activity with more frequent home experiences reflects processing efficiency, however, remains speculative given the lack of observed relation between brain activity and numerical performance. Finally, another possibility is that reduced number-specific activation in children exposed to richer home numeracy experiences could reflect reduced cognitive effort during passive viewing, or differences in processing strategy, rather than efficiency per se. Altogether, these interpretations cannot be disentangled within the current passive paradigm. Future studies combining passive and active symbolic number tasks may be able to directly test these hypotheses.

A novel finding from the present study is that home numeracy experiences (assessed through parental questionnaires and the number of children’s games at home) were positively associated with greater communication between the IPS and several brain regions. A growing body of evidence suggests that symbolic numerical processing may trigger neural communication across multiple brain regions in young children who are actively learning to associate mathematical symbols with their meanings^59^. This neural communication may be fundamental to the development of number-related brain systems. For example, longitudinal studies indicate that the connectivity of a given brain region may be associated with its subsequent specialization (e.g., related to word or letter processing^49,50,63^). Connectivity of the left IPS in children has also been found to be predictive of later numeracy skills^64,65^. Although our study is the first to show an association between home numeracy experiences and number-related brain connectivity, previous studies have suggested that brain connectivity typically increases with number knowledge, as indexed by age and enhanced symbolic knowledge^28,33,59,64^. A previous study also demonstrated an increase in brain connectivity following an intervention targeting numerical skills^66^. Overall, our findings suggest that home numeracy experiences may play a significant role in shaping the connectivity of the left IPS, which might later contribute to its specialization for digit processing. This is broadly consistent with research on the effects of home literacy experiences, which have been found to be associated with children’s language-related brain connectivity^13,28,67^.

Interestingly, we found positive associations between brain connectivity with the IPS and the frequency of both formal and informal home numeracy practices, despite the fact that only advanced formal practices were associated with children’s skills. Not only does this suggest that neural measures may be more sensitive to individual differences in experiences than behavioral measures^68^, but it also suggests that all types of numeracy practices may affect the brain system supporting numerical development in young children. However, the brain network associated with each type of practice was not homogeneous. Specifically, while informal home numeracy activities were associated with higher connectivity between the IPS and a bilateral system composed of the fusiform, precentral, and precuneus gyri, basic formal numeracy practices were associated with a right-lateralized network that included the IPS, the inferior frontal, and the supramarginal gyri. In contrast, advanced formal numeracy practices were associated with a more spatially restricted pattern of connectivity, primarily involving the bilateral cuneus. Although both basic and advanced formal practices were associated with increased connectivity in occipital regions, the underlying neural patterns were nonetheless distinct. Specifically, connectivity related to basic formal practices involved a broader right-lateralized network extending into frontal and parietal cortices, while advanced formal practices were linked to more focal effects in the bilateral cuneus (see Table 2 for anatomical details).

Thus, there appears to be a reduction in network density as we move from informal to advanced formal practices, perhaps due to differences in network maturity^64,69^ or in the range of activities that underlie informal compared to formal practices (the former being associated with a broader range of experiences and learning opportunities than the latter).

Although it is always difficult to speculate on the role of different brain regions, we note that several of the regions found in the connectivity analyses have been found critical to numerical cognition. For example, the right IPS^33,59,64,65^ and the fusiform gyrus^64,70^ may reflect the spatial and visual processing of quantitative information, respectively. It has been suggested that the cuneus may have a role in episodic memory^71^, while the supramarginal gyrus may reflect associations between digits and the quantities they represent^59^. Finally, the precentral gyrus has often been linked to finger representations, which are intertwined with the representation of numbers in young children^59,72,73^.

Finally, while PPI analyses revealed increased connectivity between the IPS and several frontal and temporal regions as a function of the home numeracy environment, these regions did not spatially overlap with those showing decreased number-specific activation. This dissociation suggests that changes in activation and connectivity may reflect distinct but potentially complementary neural mechanisms shaped by early numeracy experiences. Although we cannot exclude the possibility of functional interactions—especially given the spatial proximity between some of these regions—future studies using methods such as mediation or longitudinal designs will be needed to clarify the temporal and mechanistic links between connectivity and regional activation.

We believe that our study makes a critical contribution to the literature on the relation between numerical development and early home experiences by suggesting underlying neural mechanisms. However, we also note six main limitations of this work. First, although we argue that higher connectivity between several brain regions and the left IPS leads to a local decrease in processing demands (which might explain a decrease in brain activity), our cross-sectional design does not allow us to assess whether connectivity precedes activity or the other way around. This calls for future studies using longitudinal designs. More generally, it is important to keep in mind that the cross-sectional nature of our study limits our ability to draw any causal conclusions about the observed relations.

Second, while we showed that the frequency of home numeracy activities was related to both brain activity and connectivity with the left IPS, our results in terms of number talk are less clear. Indeed, while our measure of number talk was associated with brain activity, we did not find any relation with brain connectivity. We also failed to find any relation between number talk and children’s numerical skills, in line with several other previous studies^74–76^. A recent meta-analysis^1^ indicated a very small effect size for the relation between mathematical language used by parents and children’s mathematical skills (*r* =.03). Therefore, our sample size may have been insufficient to detect such an effect. It is also possible that our lab-based assessment of number talk may not have accurately captured the extent of the verbal interactions between parents and children^76^. Future studies should include larger samples, as well as more ecological measures of language, to better understand the relation between number talk and both skills and brain functioning.

Third, we did not find any correlation between children’s math skills and either measures of brain activity or connectivity, preventing us from investigating whether brain activity or brain connectivity mediated any relation between home numeracy experiences and skills and, therefore, from determining the functional relevance of these neural differences. This may seem relatively surprising given that such brain-behavior correlations have been found in older children and adults during active tasks requiring semantic processing of symbolic numerical information^32,37^, as well as in adaptation tasks^24,77^. Although it is always difficult to discuss a null finding, one potential explanation lies in the passive nature of our task. Indeed, while there might be a relation between automatic processing of numerical symbols (i.e., in the absence of any explicit task) and mathematical performance in older children^61^, this might be less clear in younger children (first or second grade^78^). This hypothesis is supported by findings from a study that measured brain activity associated with the implicit processing of numerical information in children aged 4 to 11 years while they watched educational videos^79^. The authors found that brain activity was linked to mathematical skills only when neural activity patterns were sufficiently mature (i.e., exhibiting more adult-like brain responses). Therefore, it is possible that any relation between brain functioning and math skills requires a level of processing of numerical information that is sufficiently mature (e.g., involving a deeper understanding of ordinality and cardinality). In addition, although the passive viewing task was designed to elicit automatic, stimulus-driven responses with minimal cognitive demands, we cannot be certain that all children engaged deeply with the symbolic stimuli. Reduced activation among children exposed to richer home numeracy environments may therefore partly reflect shallower or less consistent attention. Future studies combining passive and active number tasks could help disentangle the roles of attention, task demands, and experience-dependent neural tuning.

Fourth, although our spatial normalization procedure followed practices commonly used in developmental fMRI studies^16,50,80^, we used the standard adult MNI template rather than an age-specific brain template. This choice was motivated by the desire to maintain consistency with our previous study in 8-year-olds^24^, and to enable cross-age comparisons. However, we acknowledge that using an adult template may introduce some imprecision in anatomical localization in young children’s brains. Future studies may benefit from the use of age-appropriate pediatric templates^81,82^, which could improve spatial accuracy and individual alignment, especially in regions with high morphological variability during early childhood.

Fifth, our reliance on group-level whole-brain analyses does not account for inter-individual variability in the precise spatial location of digit-selective regions (including within the IPS) in young children. A probabilistic overlap map (Supplementary Fig. 4) illustrates substantial variability across children in the voxel-level location of peak activation for the Digits > Letters contrast, with a maximum overlap of 12 participants at any given voxel. Such heterogeneity may reduce sensitivity in cluster-level group analyses and limit the detection of brain–behavior associations. Future studies may benefit from subject-specific functional ROIs to better capture individual functional organization.

Finally, all of our participants similarly benefited from two years of public preschool education in France prior entering kindergarten. Although this ensured some homogeneity in terms of quantity of preschool experience, it does not completely control for potential differences in the quality of this preschool experience. To some extent, this is minimized by the fact that all French preschool teachers follow the standardized French preschool curriculum, However, we cannot rule out the possibility that the way this curriculum is implemented varies across schools and teachers, such that children may still differ in the extent to which they have been exposed to similar early numeracy instruction prior to our study.

In sum, the present study demonstrates the relation between early home numeracy experiences and the development of brain networks involved in number processing in young children. Our findings suggest that while the left IPS is already engaged in digit processing by five years of age, differences in home numeracy experiences may contribute not only to variations in brain activity but also to enhanced connectivity across number-related regions. We argue that the association between home numeracy experiences and number-related brain activity might reflect experience-dependent tuning and greater processing efficiency, underscoring the potential role of early numerical experiences in shaping neural pathways. While our findings are correlational in nature, they provide a foundation for future experimental studies assessing the impact of early interventions targeting the home numeracy environment of young children to enhance foundational number skills.

## Material and methods

### Participants

Participants were drawn from the 128 children of approximately 5 years of age who took part in the behavioral evaluation reported in Girard et al.^17^. The sample, which was recruited via flyers sent to schools and advertisements on social media, was mainly composed of children in kindergarten (only two children were in pre-K (i.e., second year of preschool in the French system) and eight children were within the first four months of first grade). In France, preschool education is free, starts at 3 years of age, and is attended by the vast majority of children. Two years of preschool and one year of kindergarten are organized in nursery schools (i.e., “école maternelle”), which are mandatory since 2019 but were already attended by 97.5% of children before 2018^83^.

Although all children were invited to participate in the fMRI experiment, only 106 came back to the lab for fMRI scanning. Of the children who came back for the fMRI session, 69 children did not have at least one usable run of digits and one usable run of letters for fMRI data analyses because of incomplete data (*n* = 49), excessive motion in the scanner (*n* = 17, details below), and poor performance on the target detection task embedded in the fMRI runs (*n* = 3). Therefore, 37 children were included in the final fMRI analyses. Demographic information is described in Table 3. All children and parents were native French speakers. Parents completed a questionnaire evaluating their approximate monthly income and level of education. Monthly parental income of the final sample, measured using ranges with increments of €1000, varied from less than €1000 (*n* = 5) to more than €5000 (*n* = 2) (in France, the median monthly income is about €1700^84^). Parental level of education ranged from a high school degree or below (*n* = 5) to a master’s degree or higher (*n* = 18). Therefore, socio-economic status (SES) ranged from relatively low to relatively high (see Table 3). The study was approved by a French ethics committee (Comité de Protection des Personnes du Sud-Ouest et Outre-Mer 4, reference number: CPP18-050/2018-A01229-46). Parents gave written informed consent, and children gave their oral consent to participate in the study. Families were paid 40 euros for their participation in the fMRI session.

**Table 3:** Demographic information and test scores for children and parents

Variable	Children	Parents
Age (in years)	5.79 (0.42)	37.97 (4.66)
Sex (female/male)	15/22	33/4
Parental education (number of years after high school)	-	3.82 (2.56)
Approximate monthly income (in euros)^1^	-	2257 (1234)
IQ composite (standard score)^2^	109 (15.92)	-

Values except sex are means (standard deviations are given in parentheses). 32 children were in kindergarten, 2 were in pre-K and 3 were within the first four months of first grade.

^1^ As a reference, the median monthly income in France is about €1,700 (Robin, 2019).

^2^ Scale mean = 100, Scale SD = 15.

### Pre-registration and justification of sample size

Hypotheses, sample size, methods, and planned analyses were pre-registered via the Open Science Framework (https://osf.io/yjzqn). We planned a priori to recruit a sample of 120 participants, anticipating an exclusion rate of about 60% for the fMRI analyses given the challenges raised by task-based imaging with young children. This sample size was based on analyses assuming a small to medium effect size, observed in (i) the only available meta-analysis at the time of the pre-registration about the relation between home numeracy practices and children’s numeracy skills^27^ (*r =0*.46), (ii) two studies investigating the relation between home literacy practices and language-related brain activity in young children^16,85^ (from *r* = 0.40 to *r* = 0.58), and (iii) cross-sectional neuroimaging studies of the relation between arithmetic fluency and brain activity^32,80^ (from *r* = 0.30 to *r* = 0.58). Although our exclusion rate was greater than 60%, a power analysis using G*Power 3.1^86^ indicates that our final sample of 37 participants would still give us 80% power to detect an effect size of *r* = 0.38 (at *α* = 0.05, one-tailed), which is within the anticipated range of effect sizes.

Three changes were made to the current manuscript compared to the pre-registration. First, because our sample included children who scored in the superior range on the IQ measure, we also included children whose standardized IQ was below the 25th percentile, not to bias the sample towards high IQ children (only one child who scored below the 3^rd^ percentile was excluded). Second, following our previous neuroimaging study with 8-year-olds^24^, we had planned to investigate associations between parental variables and brain activity associated with neural adaptation to repeated digits, letters, and dot arrays (i.e., a form of intra-category discrimination). However, based on pre-registered whole-brain, group-level analyses, we failed to detect significant average neural adaptation effects in any of the conditions and brain regions across participants. This may be due to methodological challenges with data acquisition (i.e., lower signal-to-noise ratio in 5-year-olds compared to older age groups^87,88^) or to a lesser degree of automaticity in number (and letter) processing in 5-year-olds compared to 8-year-olds^61^ (see also the “Discussion” section). This lack of adaptation effects across all children would have made investigating associations between individual differences in adaptation effects and other parental variables difficult to interpret. As in other studies focusing on children of equivalent age^50^, we instead focused on a form of inter-category discrimination that relies less on automatic processing. Specifically, we compared the average activity associated with digits (collapsed across blocks, see below) to the average activity associated with letters (also collapsed across blocks). Because we did not have a control condition for dot arrays (in the same way as letters are a control condition for digits because both are abstract symbols), the present report exclusively focuses on analyzing differences between activity associated with digits and with letters. Third, all of the hypotheses stated in the Introduction follow our pre-registered hypotheses, with the exception of Hypothesis #4 that originally involved both symbolic and non-symbolic processing (i.e., dot arrays). Because we ended up focusing only on symbolic processing only (see above), we are only presenting the part of the hypothesis that involves the relation between digit-related activity and the home numeracy environment. Finally, because our current analyses focused on the contrast between digits and letters (rather than on adaptation effects within each category), Hypothesis #5 from the pre-registration (initially designed to test the specificity of home numeracy effects for digit-related adaptation) became conceptually redundant with Hypothesis #3. Although the hypothesis was kept in the manuscript for transparency, we note that it no longer represents an independent analytical step (as its content is now captured by the contrast between digits and letters).

### Child measures

Numeracy and cognitive skills of all children were measured in a behavioral session prior to fMRI scanning, as detailed in Girard et al.^17^. Numeracy skills of children were assessed using the TEDI-MATH battery^89^, which includes 26 subtests assessing 7 specific numeracy skills. These are described in detail in Girard et al.^17^. Briefly, non-symbolic skills were estimated using subtests involving non-symbolic quantity comparison. Knowledge of the verbal numerical sequence was measured using subtests asking children to state the verbal numerical sequence as far as possible, as well as with lower and upper limits. Counting was assessed using subtests in which children enumerated items. Knowledge of the written number system and knowledge of the oral number system were each assessed using different subtests involving numerical decision, comparison, estimation, and judgment of grammaticality. Transcoding was estimated using two subtests involving writing and reading Arabic numbers. Calculation was assessed using eight different subtests in which children solved different types of arithmetic problems with and without visual support. We also asked parents to provide a subjective assessment of their child’s numeracy skills using a point rating scale from −2 to 2 (i.e., Not sure or no opinion was coded as 0, Severe difficulty was coded as −2, Difficulty was coded as −1, Average skills was coded as 0.5, Good skills was coded as 1 and Excellent skills was coded as 2).

In addition, a standardized full-scale IQ score was estimated for each child with the NEMI-2^90^, using verbal intelligence subtests (including general knowledge, vocabulary, and comparison) and matrix reasoning. Standard IQ scores varied from below average (min = 76) to very superior (max = 146), with a mean in the average range (see Table 1).

To ensure that children included in the fMRI sample understood the quantitative meaning of the digits presented during the fMRI task, we examined their performance on several subtasks of the NEMI-2. On the symbolic number comparison subtask, performance was very high (mean = 3.84 out of 4), with most children scoring 4/4, three scoring 3/4, and only one scoring 1/4 (note that re-running all analyses excluding this participant did not change any results). In addition, all children obtained a perfect score (8/8) on the written numerical decision subtask, confirming that they could reliably distinguish digits from letters and other non-alphanumeric symbols. Together, these findings indicate that all children included in the fMRI sample understood the quantities symbolized by single-digit numbers.

### Parental measures

Parents’ math skills, home practices, and number talk were assessed during a behavioral session prior to children’s fMRI scanning^17^. First, arithmetic skills were measured using the Math Fluency subtest of the Woodcock-Johnson-III Tests of Achievement (WJ-III^91^). Parents were asked to complete simple addition, subtraction, and multiplication problems within a 3-minute time limit. The test consists of 2 pages of 80 problems with operands ranging from 0 to 10.

Second, home numeracy practices were measured using an electronic, parent-administered questionnaire (see the list of practices in Figs. 1 and 2). To reduce the math-focus of the study, items on home numeracy practices were presented alongside items related to other types of practices (e.g., literacy, see Tables 4 and 5). For each activity, parents were asked to indicate the frequency of its occurrence at home during the previous month, using a six-point rating scale (i.e., Did not occur/Activity is not relevant to my child, 1–3 times per month, Once per week, 2–4 times per week, Almost daily, Daily). The one-month recall period, which is standard in HNE questionnaires^19^, was selected to ensure comparability with prior work and was adapted directly from the wording used in the original LeFevre et al. questionnaire^92^. Items were also adapted from the original questionnaire used by LeFevre et al.^92^ and from one of our previous studies with French elementary school children^41^. Following recommendations on a priori classifications^93,94^ and our pre-registered method, we identified 15 activities as *informal* and 21 as *formal*. Based on the pre-registration and previous studies that highlighted the importance of dissociating formal activities according to their level of difficulty^21,41^, we further identified 10 formal activities as *basic* (i.e., meeting the expectations of the French kindergarten curriculum) and 11 activities as *advanced* (i.e., exceeding the expectations of the French kindergarten curriculum). Tables 4 and 5 show how literacy activities were also divided into formal versus informal and basic versus advanced. Finally, though that measure was exploratory and not pre-registered, we also asked parents to estimate the number of games (as well as books) that they had at home.

**Table 4:** Frequency ratings associated with informal home literacy practices

Item	Mean (SD)	Min	Max
Telling invented stories	1.68 (1.51)	0	5
Talking about school day	4.49 (0.69)	3	5
Playing computer/tablet games involving reading or spelling	0.41 (0.76)	0	3
Reading texts in everyday life (advertisements, etc.)	3.27 (1.63)	0	5
Visiting the library for children’s books	0.89 (0.81)	0	2
Singing alphabet songs	1.08 (1.40)	0	5
*Average*	*1.97 (0.59)*		

Minimum rating is 0, maximum rating is 5.

**Table 5:** Frequency ratings associated with formal home literacy practices

Skill level	Item	Mean (SD)	Min	Max
Basic	Writing his/her own first name	2.51 (1.63)	0	5
	Recognizing and reading all letters	2.57 (1.72)	0	5
	Reading together	3.05 (1.60)	0	5
	*Average*	*2.71 (1.32)*		
Advanced	Reading words	2.19 (1.91)	0	5
	Reading sentences/short texts	1.42 (1.66)	0	5
	Writing words	1.86 (1.58)	0	5
	Teaching and correcting spelling	0.70 (1.37)	0	5
	Writing sentences	0.73 (1.19)	0	5
	Writing letters or stories	0.59 (1.21)	0	5
	Asking questions when we read together	3.05 (1.47)	0	5
	*Average*	1.58 (*1.22)*		

Minimum rating is 0, maximum rating is 5.

Third, parental number talk was measured during a free-play session in the lab. Each parent was given the opportunity to engage with their child in a separate room of the lab, where their dialogue and interactions were captured on video for ten minutes. Parents were instructed to interact and play with their child as they normally would at home for ten minutes. No additional instructions or prompts were given, in order to elicit naturalistic parent–child interactions. The environment was equipped with a selection of objects and toys, including picture books, play food and dishes, a cash register with pretend money, paper and colored pencils, puppets, a building game, and a set of toy vehicles^17^. The video footage was transcribed verbatim by research assistants. A random sample of 22% of the transcripts (drawn from the original behavioral sample, *n* = 120) was independently re-examined by a second assistant for transcription accuracy, yielding an agreement rate exceeding 95%. These transcriptions were then coded at the word level for instances of number talk. A subsample of 25% of the coded transcripts (*n* = 31) was double-coded by another assistant, yielding an inter-coding agreement rate above 98%. We extracted from the transcriptions every instance of number words used by the parent throughout the ten-minute play interaction^40,95^. We then calculated for each dyad the proportion of number words used by the parent (including uses of zero) out of the total number of words. We also derived additional scores for the proportion of small (0–5), medium (6–10), and large number words (more than 10) used by the parent.

### Experimental task

**Fig. 6:**
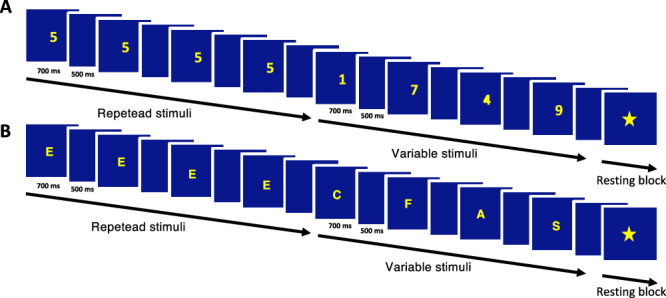
Experimental task. Participants were sequentially presented with digits (**A**) or letters (**B**) that were identical (repeated stimuli) or different (variable stimuli).

In two separate fMRI runs adapted from Girard et al.^24^, children were presented with series of digits and letters that were interspersed with periods of visual fixation. Digits ranged from 1 to 8 and letters consisted of A, B, C, D, E, F, M, R, S. Series of digits and letters were presented in blocks, half of which included eight repetitions of the same stimulus (digit or letter) whereas the other half included different stimuli (digit or letter) (see Fig. 6). This repetition manipulation was initially included to allow for the investigation of neural adaptation effects, as in our previous study^24^. Periods of visual fixation between these blocks consisted of a picture of a star that was presented at the center of the screen. In each block of digits and letters, the stimuli (digits or letters) remained on the screen for 700 ms, with a 500 ms interstimulus interval, for a total block duration of 9.6 s. Periods of visual fixation also lasted 9.6 s. In each of the two runs, there were 20 blocks of stimuli (digits or letters) and 10 blocks of visual fixation. The task was programmed and presented using PsychoPy^96^. A screen was installed at the end of the scanner bore, and stimuli were displayed by a projector in the room adjacent to the scanner. A 45° mirror was placed over the headrest so that participants could view the screen by looking upward. Head movement was minimized during the scan using cushions placed around the participant’s head. Some children were also presented with series of dot arrays in another run, which is not analyzed here for reasons detailed above.

Because no active task was asked to participants upon perceiving digits and letters, we included a target detection task to ensure that participants paid minimal attention to the stimuli. The target was the picture of a rocket that appeared randomly 10 times during the periods of visual fixation. Participants were asked to press a button every time it appeared. Participants who detected less than 3 targets in both runs were excluded from further analysis. Due to technical issues, target detection data were not recorded but instead monitored online during acquisition for 6 participants. In the final sample of children included in the fMRI analyses, the average target detection rate was 81% (*SD* = 21) in the digit run and 82% (*SD* = 19) in the letter run. No difference in target detection rate was observed between the runs (Wilcoxon rank sum test: *p* = 0.71), indicating that children paid equal attention to the stimuli in both runs.

### fMRI data acquisition

To ensure children’s comfort and optimize data quality, we implemented a child-adapted MRI protocol. Before scanning, children and parents viewed a short video introducing both the mock and real MRI environments. During a preparatory session in an inflatable mock scanner, children practiced the task, heard scanner sounds, and were instructed to remain still using real-time motion feedback. The actual MRI room was decorated as a spaceship to create a child-friendly environment, and a pediatric head coil with inflatable pads was used to maximize comfort and head stability. Active noise-canceling headphones, real-time motion tracking (FIRMM Software^97^), and an in-coil camera allowed continuous monitoring of gaze and movements. During the anatomical sequence, children watched a cartoon movie to maintain comfort and reduce motion.

Images were collected with a Siemens Prisma 3 T MRI scanner (Siemens Healthcare, Erlangen, Germany) at the CERMEP Imagerie du vivant in Lyon, France. The BOLD signal was measured with a susceptibility weighted single-shot EPI sequence. Imaging parameters were as follows: TR = 2000 ms, TE = 24 ms, flip angle = 80°, matrix size = 128 × 120, field of view = 220 × 206 mm, slice thickness = 3 mm (0.48 mm gap), number of slices = 32. A high-resolution T1-weighted whole-brain anatomical volume was also collected for each participant. Parameters were as follows: TR = 3500 ms, TE = 2.24 ms, flip angle = 8°, matrix size = 256 × 256, field of view = 224 × 224 mm, slice thickness = 0.9 mm, number of slices = 192.

### fMRI data preprocessing

Using the SPM software (https://www.fil.ion.ucl.ac.uk/spm/), functional images were corrected for slice acquisition delays and for spatial realignment to the first image of the first run to account for head motion. Realigned images were smoothed with a Gaussian filter (4×4×7 mm full-width at half maximum). Motion outliers were detected using ArtRepair (https://www.nitrc.org/projects/art_repair/), in accordance with our pre-registered data processing plan (https://osf.io/yjzqn) and consistent with our previous studies^15,24^. Volumes were flagged as outliers if they exhibited (i) a global mean intensity greater than 3 standard deviations from the run average or (ii) volume-to-volume motion greater than 2 mm. These outlier volumes were replaced by interpolating the two closest non-flagged volumes. Participants were excluded from the analyses if more than 20% of the volumes were repaired in either the digit or the letter runs (*n* = 17). For these excluded participants, the average percentage of repaired volumes was 47% for the digit task (range = 20.4–90.8%) and 64% for the letter task (range = 22.4–98%). To verify that excluding these participants did not bias our sample, we compared home numeracy measures and children’s numeracy skills between included and excluded participants (based on motion-related exclusion) using independent samples t-tests. No significant group differences were found (all *t*s < 1.55, all *p*s > 0.13). The proportion of repaired volumes for each included participant, for both digit and letter runs, is reported in the shared behavioral dataset.

Finally, functional images were normalized into the standard Montreal Neurological Institute (MNI) space, consistent with previous developmental fMRI studies in preschool children^16,50,80^. This approach also ensured comparability with our prior study in 8-year-olds^24^. This was done in two steps. First, after coregistration with the functional data, the structural image was segmented into gray matter, white matter, and cerebrospinal fluid by using a unified segmentation algorithm^98^. Second, the functional data were normalized to the MNI space by using the normalization parameters estimated during unified segmentation (normalized voxel size, 2 mm^3^ × 2 mm^3^ × 3.5 mm^3^).

### fMRI data analysis

Statistical analysis of the fMRI data was performed with a general linear model implemented in the SPM software. At the individual level, brain activity associated with blocks of digits and letters was modeled as epochs with onsets time-locked to the beginning of each block and a duration of 9.6 s, with separate regressors for series of identical and different stimuli. Blocks of visual fixation were also modeled in a separate regressor. All epochs were convolved with a canonical hemodynamic response function. The time series data were high-pass filtered (1/128 Hz), and serial correlations were corrected with an auto-regressive AR(1) model.

At the individual level, blocks were modeled separately according to both the stimulus category (digits vs. letters) and the repetition condition (repeated vs. variable), to examine potential adaptation effects (consistent with our pre-registered plan). However, since no significant adaptation effect was observed in any condition or brain region (see “Materials and Methods” Section “Pre-registration and justification of sample size”), we pooled across repetition conditions in our contrasts of interest. Specifically, we used contrast weights that combined both repetition conditions within each stimulus category (e.g., [1 1 −1 −1] to contrast digits vs. letters, pooling across repetition conditions). This approach effectively examines digit-related versus letter-related activity while accounting for any potential repetition effects in the model. Such an inter-category discrimination approach has been successfully used in prior developmental neuroimaging studies^50^.

Brain activity associated with blocks of digits and letters was modeled separately. Blocks of visual fixation were also modeled explicitly. For each subject, brain activity associated with all blocks of digits (versus fixation) was contrasted to brain activity associated with all blocks of letters (versus fixation) to capture activity that was specifically elicited by perceiving digits. This contrast reflects regions that respond more to digits than to letters, while the reverse contrast (Letters > Digits) captures regions preferentially activated during letter processing. Subject-level contrasts were then entered into 2nd-level.

In line with our pre-registered hypotheses, we conducted five independent whole-brain regression analyses, each including a home environment predictor: (1) informal home numeracy practices, (2) basic formal practices, (3) advanced formal practices, (4) the number of children’s games at home, and (5) the proportion of parental number words.

All models were implemented as whole-brain voxelwise analyses, consistent with our pre-registered analytic plan. All statistical maps were thresholded at a cluster-forming threshold of *p* < 0.0025 (uncorrected) and corrected for multiple comparisons at the cluster level using a family-wise error (FWE) correction of *p* < 0.05, consistent with our previous study^24^. For transparency and comparability with other studies, we also report in the figures and tables the clusters that survive a FWE correction (*p* < 0.05) using a more conservative cluster-forming threshold of *p* < 0.001 (uncorrected).

In addition, exploratory analyses were performed using psychophysiological interaction (PPI) analyses to assess task-dependent changes in functional connectivity^99,100^. PPI analyses estimate whether the functional coupling between a seed region and the rest of the brain is modulated by a psychological condition (here, digits vs. letters). This method has been widely used in cognitive neuroimaging^101,102^, including in studies on numerical cognition with children^59,80,103^.

A PPI analysis assesses whether certain brain areas (target regions) show activity that can be explained in terms of an interaction between the influence of a distal area (source region) and an experimental parameter. In other words, a PPI analysis tests whether activity in a source area contributes to activity in a target area to a greater (or lesser) extent in one condition versus another. Here, we aimed to explore how functional connectivity between the IPS and other brain regions changed as a function of home numeracy practices, parental number talk, and children’s numeracy skills when children processed digits (as compared to letters). At the first level, the PPI model included three regressors: (i) the average time series of the IPS (i.e., the “physiological” part of the PPI), (ii) the contrast of digits versus letters after it had been convolved with a standard HRF (the “psychological” parts of the PPI), and (iii) the interaction between the physiological and psychological factors (i.e., the “interaction” part of the PPI). The average time series of the IPS was defined as the first eigenvariate time series extracted from a 6 mm radius sphere centered around the IPS coordinates in the contrast of digits versus letters across all participants. To compute the interaction regressor, the BOLD signal from the IPS was deconvolved by using a Bayesian estimation algorithm^8^. The regressor coding for the contrast of digits versus letters was multiplied by the deconvolved seed activity regressor to produce the interaction term. This interaction term, which was in the end convolved with a standard HRF, quantified the degree to which activity in various brain regions may be explained by the interaction between IPS activity and a difference between perceiving digits versus letters. Individual whole-brain beta maps of the interaction term were entered into 2nd-level regression analyses that included as predictors home numeracy practices, parental number talk, number of games at home, and children’s numeracy skills. Consistent with our pre-registration, all PPI analyses were also conducted at the whole-brain level, without applying any region-of-interest or small-volume corrections. A cluster-level threshold of *p* < 0.05 (defined using a cluster-forming threshold of *p* < 0.0025) corrected for family wise error was applied to all whole-brain statistical maps to assess brain areas of significant connectivity changes with the IPS. Because most of the analyses involved brain-behavior correlations, Cook’s distances were systematically examined for all activated clusters, and outliers were identified and removed if Cook’s distance > 1 (this was the case for one analysis on the proportion of parental number words; see Results).

## Supplementary information

Supplementary information

## Data Availability

The pre-registration, questionnaires, fMRI task, behavioral data, and individual beta maps that support the findings of this study are available via the OSF at https://osf.io/vuar8/.

## Code availability

The Matlab scripts used to analyze the fMRI data are available via the OSF at https://osf.io/vuar8. The scripts are also available on GitHub (https://github.com/BBL-lab/BBL-batch-system).
