# Supplementary material for: Home numeracy experiences are associated with number-related brain activity and connectivity in early childhood
Source: NPJ Sci Learn. 2026 Apr 3;11:31. doi: 10.1038/s41539-026-00419-5 (PMC13223258; doi:10.1038/s41539-026-00419-5)
Supplement: Supplementary file 1 — Supplementary information [file 41539_2026_419_MOESM1_ESM.docx]

**Supplementary Information**

***Results***

***Behavioral associations between children’s numeracy skills and home numeracy practices***

| **Table S1.** Correlation matrix between home numeracy practices and children's numeracy skills in the fMRI subsample (*n* = 37). | | | | | | | | | | |
| --- | --- | --- | --- | --- | --- | --- | --- | --- | --- | --- |
|  | **1.** | **2.** | **3.** | **4.** | **5.** | **6.** | **7.** | **8.** | **9.** | **10.** |
|  |  |  |  |  |  |  |  |  |  |  |
| ***Home numeracy practices*** |  |  |  |  |  |  |  |  |  |  |
| **1. Informal** | - |  |  |  |  |  |  |  |  |  |
| **2. Formal / basic** | 0.43** | - |  |  |  |  |  |  |  |  |
| **3. Formal / advanced** | 0.42** | **0.65***** | - |  |  |  |  |  |  |  |
|  |  |  |  |  |  |  |  |  |  |  |
| ***TEDI-math*** |  |  |  |  |  |  |  |  |  |  |
| **4. Non-symbolic skills** | 0.28 | -0.07 | 0.38* | - |  |  |  |  |  |  |
| **5. Verbal numerical sequence** | -0.06 | -0.47** | 0.12 | 0.33* | - |  |  |  |  |  |
| **6. Counting** | 0.20 | -0.14 | 0.27 | 0.46* | 0.29 | - |  |  |  |  |
| **7. Written number system** | 0.07 | -0.41** | 0.06 | 0.34* | **0.67***** | 0.24 | - |  |  |  |
| **8. Oral number system** | -0.06 | -0.42** | 0.17 | 0.40* | **0.65***** | 0.30 | **0.66***** | - |  |  |
| **9. Transcoding** | 0.05 | -0.30 | **0.63***** | 0.40* | **0.52**** | 0.33* | 0.45** | 0.42** | - |  |
| **10. Calculation** | 0.15 | -0.37** | 0.40* | **0.57***** | **0.56***** | **0.53***** | **0.52***** | **0.65***** | **0.72***** | - |
|  |  |  |  |  |  |  |  |  |  |  |

**Notes**. *N*= 37, **p* < .05, ***p* < .01, ******* *p*<.001. A Bonferroni correction for multiple comparisons (45 tests) sets the significance threshold at *p* < .0011 (significant results after correction are highlighted in bold in the table). Correlations involving advanced formal practices are partial correlations controlling for basic formal practices.

| **Table S2.** Comparison of correlations between HNE factors and children’s numeracy skills across the full behavioral sample (*n* = 128) and the fMRI subsample (*n* = 37) | | | |
| --- | --- | --- | --- |
| **Predictor** | **Outcome** | **Full sample (*n* = 128)** | **fMRI subsample (*n* = 37)** |
| **Advanced formal practices** | Transcoding | *r* = .52*** | *r* = .63** |
|  | Calculation | *r* = .33** | *r* = .40* |
|  | Non-symbolic | ns | *r* = .38* |
| **Basic formal practices** | All outcomes | ns | ns |
| **Informal practices** | All outcomes | ns | ns |
| **Number of games at home** | Counting | not tested | *r* = .36* |
|  | Non-symbolic | not tested | *r* = .50** |
|  | Other outcomes | not tested | ns |
| **Parental number talk (proportion)** | All outcomes | ns | ns |

**Note**s. ns = not significant (all *p* > .05). **p* < .05, ***p* < .01, ****p<*.001

| **Table S3.** Sensitivity analyses: Partial correlations between advanced formal home numeracy practices and children's numeracy skills, controlling for child sex, age, and fMRI motion | | | | |
| --- | --- | --- | --- | --- |
| **Numeracy skill** | **Zero-order *r*** | **Partial r** | | |
|  |  | **Sex** | **Age** | ***Motion*** |
| **Non symbolic** | 0.39* | 0.35* | 0.35* | 0.39* |
| **Verbal numerical chain** | 0.12 | - | - | - |
| **Counting** | 0.27 | - | - | - |
| **Written number system** | 0.06 | - | - | - |
| **Oral number system** | 0.17 | - | - | - |
| **Transcoding** | 0.63*** | 0.61*** | 0.58*** | 0.59*** |
| **Calculation** | 0.40* | 0.36* | 0.38* | 0.36* |

**Notes**. *N*= 37, **p* < .05, ***p* < .01, ******* *p*<.001. Zero-order *r* = bivariate correlation. Partial *r* = partial correlation controlling for indicated covariate. Motion = percentage of repaired volumes separately computed for the digit and letter runs

***Relations between the home numeracy environment and number-specific activity***

**Figure S1.** Negative associations between home numeracy experiences and number-specific brain activity after controlling for cognitive and socioeconomic factors.

Surface renderings of brain regions showing negative associations between number-specific activity (digits > letters) and different aspects of the home numeracy environment, after controlling for family SES (parental education and income), children’s IQ, parental numeracy skills, and parents’ subjective estimate of their child’s numeracy level. Colors indicate statistical significance at two cluster-forming thresholds (dark blue for p < .0025, light blue for p < .001).

**Figure S2.** Negative associations between literacy-related home experiences and number-specific brain activity.

Surface renderings of brain regions showing negative associations between number-specific activity (digits > letters) and different aspects of the home literacy environment. Colors indicate statistical significance at two cluster-forming thresholds (dark blue for p < .0025, light blue for p < .001).

**Figure S3.** Scatterplots showing the associations between each aspect of the home numeracy environment and brain activity for the Digits > Rest contrast, extracted from the same functional clusters displayed in **Figure 4**. Scatterplots illustrate individual data points and regression lines.


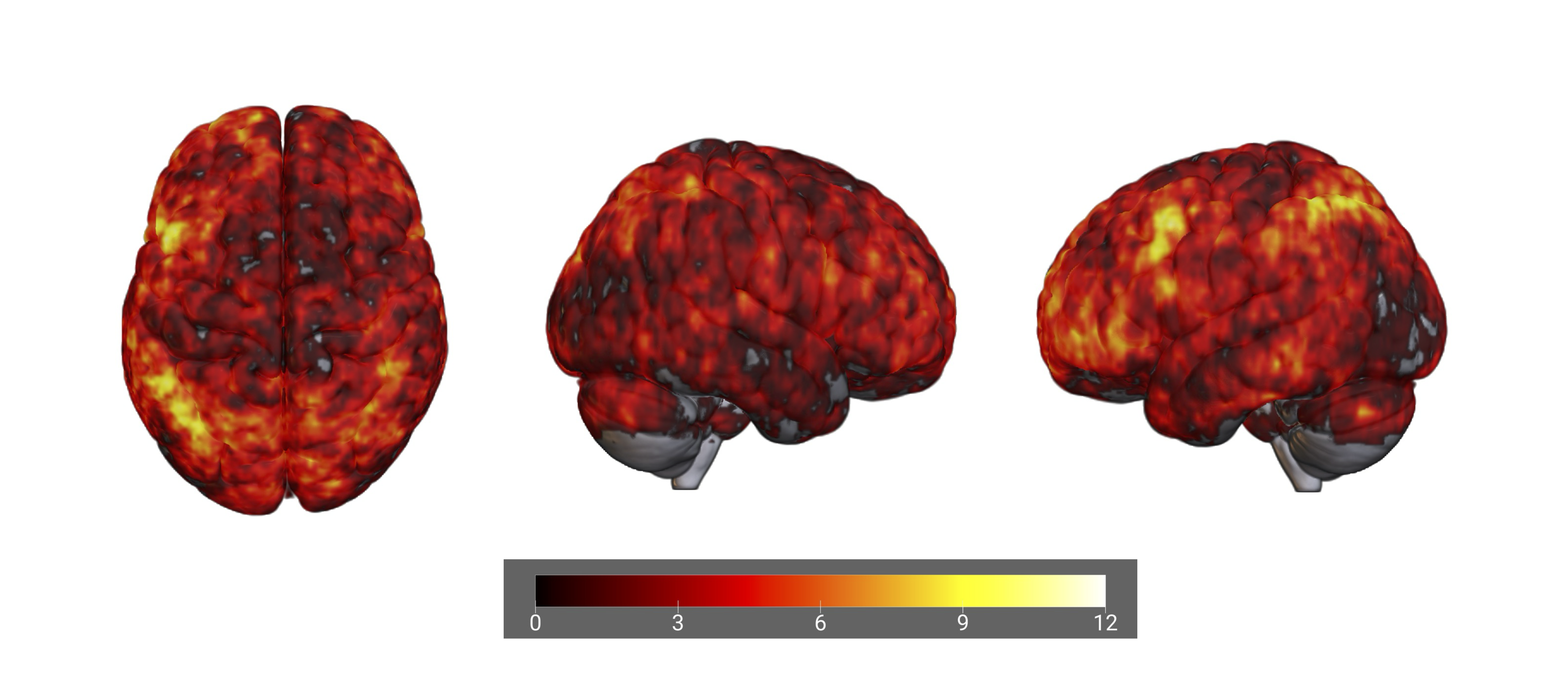


**Figure S4.** Probabilistic overlap map for the Digits > Letters contrast across participants.

Surface renderings (top, left, right views) showing how many children share peak activation in the same voxels. For each participant, the top 10% most activated voxels were binarized and overlapped (range: 0–12).
